# Patients' views on outcome following head injury: a qualitative study

**DOI:** 10.1186/1471-2296-6-30

**Published:** 2005-07-27

**Authors:** Paul Graham Morris, Lindsay Prior, Shoumitro Deb, Glyn Lewis, Wendy Mayle, Caroline E Burrow, Eleanor Bryant

**Affiliations:** 1 Section of Clinical & Health Psychology, Old Medical School, Teviot Place, Edinburgh EH8 9AG, Scotland; 2 School of Social Sciences, Cardiff University, Cardiff CF10 3WT, Wales; 3 Department of Psychiatry, University of Birmingham, B15 2QZ, England; 4 University of Bristol, Cotham House, Cotham Hill, Bristol BS6 6JL, England; 5 Headway Cardiff, Rookwood Hospital, Cardiff, Wales

## Abstract

**Background:**

Head injuries are a common occurrence, with continuing care in the years following injury being provided by primary care teams and a variety of speciality services. The literature on outcome currently reflects areas considered important by health-care professionals, though these may differ in some respects from the views of head injured individuals themselves. Our study aimed to identify aspects of outcome considered important by survivors of traumatic head injury.

**Methods:**

Thirty-two individuals were interviewed, each of whom had suffered head injury between one and ten years previously from which they still had residual difficulties. Purposive sampling was used in order to ensure that views were represented from individuals of differing age, gender and level of disability. These interviews were fully transcribed and analysed qualitatively by a psychologist, a sociologist and a psychiatrist with regular meetings to discuss the coding.

**Results:**

Aspects of outcome mentioned by head injury survivors which have received less attention previously included: specific difficulties with group conversations; changes in physical appearance due to scarring or weight change; a sense of loss for the life and sense of self that they had before the injury; and negative reactions of others, often due to lack of understanding of the consequences of injury amongst both family and general public.

**Conclusion:**

Some aspects of outcome viewed as important by survivors of head injury may be overlooked by health professionals. Consideration of these areas of outcome and the development of suitable interventions should help to improve functional outcome for patients.

## Background

The consequences of brain injury upon an individual are many and varied, with some individuals fortunately making a good recovery whilst others continue to be affected in the longer term. Residual difficulties one year post injury often include physical effects such as fatigue, paralysis, seizures and headaches and/or cognitive effects such as difficulties with attention, short-term memory, planning or language. Increasing recognition in recent years has been given to social and psychological aspects of outcome such as anxiety, depression and social isolation [1-5].

Most studies of outcome following head injury have been based upon questionnaires, which can severely constrain responses and often focus upon the presence of symptoms rather than the degree to which they are causing problems. These outcome measures are invariably devised by health care professionals, reflecting aspects of outcome that they consider important, but with little or no input from head injured individuals themselves. It is reasonable to suppose that professionals and patients will differ in some respects in the aspects of outcome that they consider important and the language used to describe these areas of outcome. However there has been little previous research aimed at eliciting the unconstrained views of head injured individuals themselves upon aspects of outcome.

Qualitative studies of outcome following stroke have enabled a greater understanding of many aspects of individuals' responses to stroke [6], including how patients recognise and respond to their stroke [7]; their experiences whilst in hospital [8]; the strategies they use to manage their illness [9] and their information needs following stroke [10,11]. One such study highlighted consequences of stroke that were important to stroke survivors, but which probably would not have been identified using standardised outcome measures [12].

In order to identify consequences of head injury considered important by patients, we conducted qualitative interviews with survivors of head injury who had residual symptoms at least one year post injury.

## Methods

### Design of study

Semi-structured interviews were conducted with 32 individuals who had suffered head injury and subsequently returned to a home environment.

### Participants

A purposive sampling strategy was employed in order to ensure that views were represented from individuals of differing age, gender and level of disability (Table 1). Potential interviewees with probable moderate or severe disability resulting from a traumatic head injury sustained whilst aged over 16 were identified via local head injury services. Actual level of disability was subsequently determined using the extended Glasgow Outcome Scale, based upon information obtained at interview [13]. Eighty-nine were contacted by letter to explain the study, of these 42 replied and 40 were willing to be interviewed about the consequences of their injury. Thirty-two of these were interviewed, by which time no further themes were being generated and the analysis was deemed to have reached saturation.

**Table 1:** Number of Participants by Age, Gender and Disability Level

Age at Injury	Gender	Upper Moderate Disability	Lower Moderate Disability	Severe Disability
16 – 29	Male	6	6	4
	Female	1	3	1
30+	Male	3	3	2
	Female	0	3	0

Disability levels are based on extended Glasgow Outcome Scale [13]

#### Ethical Approval

Approval was obtained from the local Bro Taff NHS Health Ethics committee and informed consent was given by all patients who participated in the study.

### Interviews

Those who agreed to participate were visited in their own homes and again had the opportunity to ask questions about the study before being asked to complete consent forms. All interviews were conducted at least one year post injury (range 1–10 years) and all interviewees had returned to a home environment for at least six months prior to being interviewed. Where possible the head injured individual was interviewed alone, though in two cases a carer was present. The interviews were semi-structured, with interviewees asked to describe their lives prior to the injury and then to describe the consequences of head injury that had been most important to them. These interviews were all recorded onto minidisk and transcribed in full.

#### Analysis

**Figure 1:**
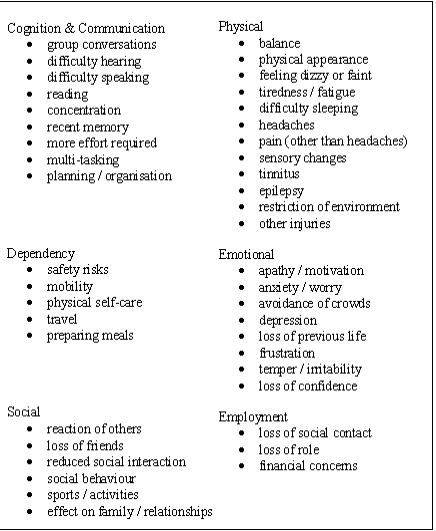
Interacting domains and categories of outcome.

The analysis was a continual process in parallel with data collection, involving the repeated reading of recent transcripts in combination with listening to the recorded interviews. Emergent themes reported by participants as being important in their outcome following head injury were then coded by a health psychologist with experience of working with brain injured individuals (PGM). A random selection of over 50% of the transcripts were read and coded separately by a sociologist (LP) and a neuropsychiatrist (SD), with regular meetings held to discuss the coding. A sampling to saturation strategy was employed, with 139 themes generated by the time of the 32^nd ^interview, by which time no further themes were emerging from new interviews. Through a process of discussion and comparison of transcripts, this list of codes was merged into 43 broader representative categories which were then grouped into six domains (Figure 1). These outcome categories were then discussed with members of a local head injury support group.

## Results

The distribution of participants by age at injury, gender and level of disability is shown in Table 1. Representative categories of outcome divided into six domains are shown in Figure 1. There is considerable interaction and overlap between these domains and they are not intended to be viewed as independent from each other. For example, difficulties with group conversations often led to reduced social interaction, which in turn is often a cause of depression. Also fatigue may be viewed as much as a cognitive or emotional problem as a physical one. The domains are thus intended as interacting and interdependent and, as would be expected, most of these categories reflect areas of outcome that are well recognised in the current literature. Our results focus upon those categories of outcome which are less well documented and less often measured in current outcome measures.

### Group conversations

**Figure 2:**
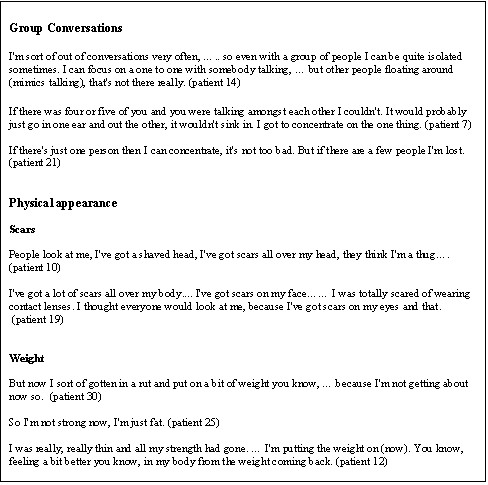
Group conversations and physical appearance.

Particular difficulties in attending to more than one voice at a time were reported by eleven participants (Figure 2). In each case the individuals concerned had no difficulties understanding a single voice, but were unable to make sense of conversations where several people were speaking at once: "I only listen to one at a time" (patient 18); "I found it very difficult to cope with two voices" (patient 14). As a result, some reported feeling isolated or uncomfortable in these situations and consequently avoided situations, such as pubs, where they were likely to have to deal with conversations involving more than one other person. This naturally resulted in less social interaction, which itself was identified by most participants as an important aspect of their outcome.

### Physical appearance

Eight participants mentioned changes in their physical appearance as important aspects of their outcome (Figure 2). These were generally either due to scars or weight change, resulting in concerns about self-image and the perceptions of others. Scars were caused either by neurosurgery or by other injuries which were sustained at the same time as the head injury. Whilst concerns about gains in weight due to less activity were mentioned by both males and females, loss of weight and strength was also mentioned by one male as an important aspect of his outcome.

### Loss

**Figure 3:**
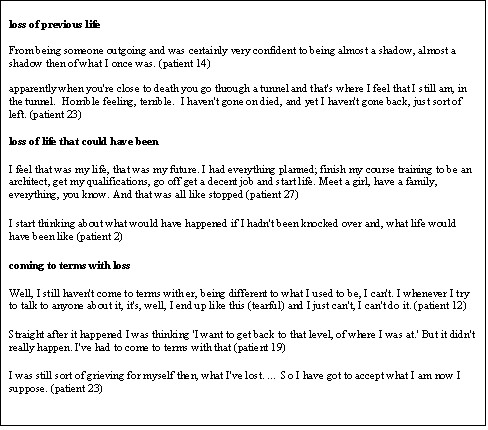
Loss.

Most participants mentioned feelings of loss relating to aspects of the life that they had before the head injury: "a head injury should have a warning of great losses," (patient 11); "I just seem to have lost everything" (patient 24) (Figure 3). These losses included loss of work, of friends, of partners and of abilities. Some felt that they had lost the person they were before the injury, feeling that they were a totally different person now: "I don't think I will ever go back to the young woman I was before the accident" (patient 23); "...it was like a different life after the accident" (patient 28). Others mentioned feelings of loss for the life that they would have had now if the head injury hadn't happened: "I start thinking about what would have happened if I hadn't been knocked over and what life would have been like..." (patient 2).

### Negative reactions of others

**Figure 4:**
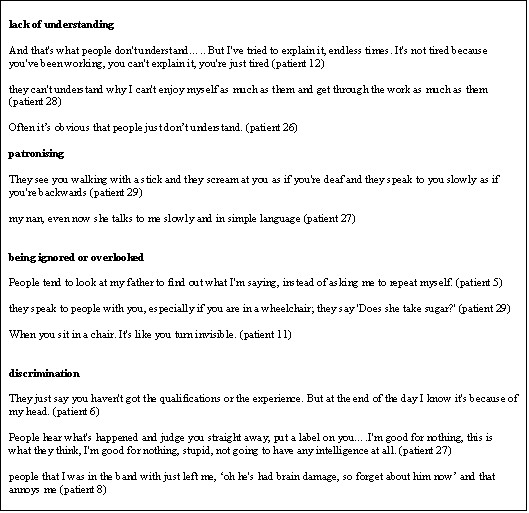
Negative reactions of others.

Sixteen participants mentioned difficulties which were directly related to the reactions of others, some examples of which are given in Figure 4. Often these negative reactions were due to a lack of understanding of the consequences of head injury, both amongst the general public and some health care professionals. Thus, for example, some people couldn't understand why head injured individuals were often depressed or exhausted. Sometimes such lack of understanding seemed in part to be due to the absence of external signs of injury. "They put a tie on you and cut your hair and you look ok don't you" (patient 14); "physically I might look alright, but mentally ..." (patient 16); "I looked exactly the same as I did before, so what's really changed is in here (pointing at head), not outside" (patient 17). Consequently, as others could see no external sign of injury they expected them to cope as well as anyone else. Others over-compensated and inadvertently caused offence by treating head injured individuals as if they were far less capable than was actually the case.

Other participants mentioned being ignored or over-looked by others. This was particularly a problem amongst those who used wheelchairs since their injury or had some speech impairment (Figure 4). Other negative reactions included discrimination which had resulted in difficulties finding suitable employment or in being excluded from social groups.

## Discussion

The results highlight areas of outcome which are important to survivors of head injury but which have previously received less attention than they perhaps deserve. This may be due in part to their being less easily identified either in clinical interviews or via current outcome measures. The areas of outcome and means of description derived from these patient interviews have subsequently been used to develop a patient centred outcome measure which focuses upon the effects that particular symptoms have upon the individual, rather than simply whether they are present.

Amongst those areas of outcome perhaps worthy of further attention, difficulties with group conversations were mentioned as an important aspect of outcome by eleven participants. However as these individuals had no difficulties understanding single voices, these difficulties might not be identified by interview or questionnaires unless they asked specifically about situations where there were several voices. Whilst cognitive difficulties with divided attention following head injury are reasonably well established [14], the translation to how this affects day-to-day life for survivors in terms of group conversations appears less well known. Although these individuals would not have any input from speech and language therapy, one individual (patient 3) mentioned that he focused on people's lips more in order to cope when there were other distracting voices. Thus it is possible that some training in lip-reading may be of benefit to individuals with difficulties following group conversations.

It is possible that concerns about physical appearance are under-reported to health care professionals, as they are viewed as cosmetic rather than 'medical'. Also males, who are several times more likely to suffer head injury, may be particularly reticent to mention such concerns. However the interviews highlight that these changes are of concern to survivors and may influence self-esteem and sense of identity, particularly amongst individuals feeling more vulnerable due to other consequences of their injury.

A sense of loss was mentioned by most interviewees. Whilst some had begun to come to terms with these losses and accept their new self and abilities, others were still unable to accept their losses and these continued to be a cause of depression and frustration for them. As such, some aspects of bereavement counselling may be beneficial in supporting individuals with head injury.

Misconceptions and negative reactions were sometimes present amongst family as well as amongst the general public, with similar misconceptions previously reported in a study of carers and health care professionals [15]. Whilst misunderstandings amongst the general public may be more difficult to address, the provision of suitable information to family and friends may help to reduce difficulties due to lack of understanding. Videos have a number of potential benefits in conveying such information, including being able to be watched by the whole family at the same time, enabling illustration of case examples by 'real' people rather than just quotes and being easier to attend to than written or verbal information.

### Strengths and limitations of this study

This study reports the views of survivors of head injury themselves, validating the importance of some areas of outcome and highlighting aspects of outcome whose importance has either been under-estimated or viewed differently by health care professionals. The sample was selected in order to obtain a range of views from individuals of differing age, gender and disability, with sampling continuing until no further themes were being generated. The study focused upon those with residual difficulties at least one year after head injury, excluding those who had made a good recovery as measured by the extended Glasgow Outcome Scale. Whilst it is appreciated that some of these individuals have residual difficulties, it is likely that these would be fewer and less pronounced and would also be present amongst some of those with moderate or severe disability and thus accounted for in our study.

All of the interviews were recorded and fully transcribed, with these interviews coded by three investigators, each of whom could bring differing relevant training and experience to facilitate the coding. This process reduces, but naturally cannot exclude, the potential for bias in the analysis of the interviews. However some validation for the findings was provided by head injury survivors who attended the local Headway group, who agreed that the key areas of outcome identified by the study accurately reflected aspects of outcome that were important following head injury.

## Conclusion

Head injury leads to a wide range of emotional, social, cognitive and physical difficulties, with primary care teams and various speciality services providing support and care to those who have returned to a home environment. Whilst most areas of outcome mentioned by survivors are already well known, areas of outcome where more attention might be deserved include: difficulties with group conversations in individuals who have no problems with one-to-one conversations, sensitivity to concerns relating to changes in physical appearance, the consideration of sense of loss amongst individuals following injury and the various misconceptions and negative reactions from others. Consideration of these aspects of outcome should help to enhance understanding of the difficulties faced by head injury survivors and improve functional outcome for these patients.

## Competing interests

The author(s) declare that they have no competing interests.

## Authors' contributions

PGM was involved in study design, conducted and analysed interviews, wrote the paper and will serve as guarantor for the integrity of the data. LP and SD were both involved in study design, analysed interviews and contributed to editing the paper. GL and WM were both involved in study design and contributed to editing the paper. CEB and EB both contributed to editing the paper.

## Pre-publication history

The pre-publication history for this paper can be accessed here:


